# Hemodialysis in Brazil: differences across geographic regions regarding demographics, laboratory parameters and drug prescription

**DOI:** 10.1590/2175-8239-JBN-2022-0169en

**Published:** 2023-06-30

**Authors:** Fabiana Baggio Nerbass, Helbert do Nascimento Lima, Jocemir Ronaldo Lugon, Ricardo Sesso, The Brazilian Dialysis Register Investigators

**Affiliations:** 1 Fundação Pró-Rim, Joinville, SC, Brazil.; 2 Universidade da Região de Joinville, Joinville, SC, Brazil.; 3 Universidade Federal Fluminense, Niterói, RJ, Brazil.; 4 Universidade Federal de São Paulo, São Paulo, SP, Brazil.

**Keywords:** Renal Dialysi, Epidemiology, Diálise Renal, Epidemiologia

## Abstract

**Introduction:**

Brazil has a vast territory divided into five geographic regions with important differences in sociodemographic indices. We aimed to present and compare socio-demographic characteristics, biochemical results, and drug prescription of patients on chronic hemodialysis (HD) treatment in the five geographic regions.

**Methods:**

We evaluated data from the Brazilian Dialysis Registry of all adult patients undergoing chronic HD in 2021. Variables included sociodemographic characteristics, serum levels of phosphate, calcium, and albumin, hemoglobin, urea reduction rate, and prescription of phosphate binders, erythropoietin, and intravenous iron. Data from the North and Northeast regions were combined into one group.

**Results:**

A total of 13,792 patients (57.9 ± 16.0 years old, 58.5% male, median HD vintage of 31 (11–66) months) from 73 dialysis centers were analyzed. Regional distribution was 59.5% in the Southeast; 21.7% in the South; 5.9% in the Midwest; and 12.9% in the North/Northeast. Sociodemographic features, biochemical results, and medication prescriptions differed across regions. The prevalence of elderly patients was lower in the Midwest and North/Northeast. The South region had the highest prevalence of hyperphosphatemia (41.2%) and urea reduction rate <65% (24.8%), while anemia and hypoalbuminemia were more prevalent in the Southeast, 32.7% and 11.6%, respectively.

**Conclusion:**

We found differences in socio-demographics, clinical features, and drug prescriptions across Brazilian geographic regions. Some findings reflect the socio-demographic diversity of the country, while others deserve further elucidation.

## Abstract (pt)

**Introdução:**

O Brasil possui um vasto território dividido em cinco regiões geográficas com importantes diferenças nos índices sociodemográficos. Nosso objetivo foi apresentar e comparar características sociodemográficas, resultados bioquímicos e prescrição de medicamentos de pacientes em tratamento de hemodiálise crônica (HD) nas cinco regiões geográficas.

**Métodos:**

Avaliamos os dados do Registro Brasileiro de Diálise de todos os pacientes adultos submetidos à HD crônica em 2021. As variáveis incluíram características sociodemográficas, níveis séricos de fosfato, cálcio e albumina, hemoglobina, taxa de redução de ureia e prescrição de quelantes de fosfato, eritropoietina e ferro intravenoso. Os dados das regiões Norte e Nordeste foram combinados em um único grupo.

**Resultados:**

Foi analisado um total de 13.792 pacientes (57,9 ± 16,0 anos, 58,5% do sexo masculino, mediana de tempo de HD de 31 (11–66) meses) de 73 centros de diálise. A distribuição regional foi de 59,5% dos pacientes provenientes do Sudeste; 21,7% do Sul; 5,9% do Centro-Oeste; e 12,9% do Norte/Nordeste. As características sociodemográficas, os resultados bioquímicos e as prescrições de medicamentos diferiram entre as regiões. A prevalência de pacientes idosos foi menor nas regiões Centro-Oeste e Norte/Nordeste. A região Sul apresentou a maior prevalência de hiperfosfatemia (41,2%) e taxa de redução de ureia < 65% (24,8%), enquanto a anemia e a hipoalbuminemia foram mais prevalentes no Sudeste, 32,7% e 11,6%, respectivamente.

**Conclusão:**

Encontramos diferenças nos dados sociodemográficos, nas características clínicas e prescrições de medicamentos nas regiões geográficas brasileiras. Alguns achados refletem a diversidade sociodemográfica do país, enquanto outros demandam maiores esclarecimentos.

## Introduction

Brazil has a population of more than 213 million people and a land area of over 8.5 million km^2 1^. The vast Brazilian territory is divided into five geographic regions that differ in demographic, cultural, social, economic, and health aspects^2^.

It was estimated that almost 150 thousand Brazilians were on chronic dialysis treatment in 2021, 94.2% of them on hemodialysis^3^. Significant regional differences in demographic, clinical, and outcomes were found in a large Brazilian cohort of peritoneal dialysis patients followed from 2004–2007^4^, but little is known about the current profile of people on HD in the different regions.

The identification of possible regional differences in socio-demographic and laboratory parameters among the growing number of patients on chronic HD can expand the understanding of possible barriers and regional characteristics related to HD treatment. Using data from a large Brazilian cohort of chronic dialysis, we presented and compared demographic characteristics, biochemical results, and drug prescription across geographical regions.

## Methods

This is a retrospective analysis of data from the Brazilian Dialysis Registry (BDR), a non-probabilistic national electronic database of standardized clinical and epidemiological information (baseline and follow-up) of patients undergoing dialysis. Detailed methods for the BDR data collection have been published elsewhere^5^.

Patients from 73 dialysis centers were included in this analysis, corresponding to 9% of the total number of Brazilian centers (14% in the South, 9% in the Southeast and Midwest, 8% in the North, and 3% in the Northeast).

In this study, we evaluated data of all incident and prevalent adult patients (>18 years old) undergoing chronic HD in 2021. We excluded participants with less than two months of information in 2021 and patients on peritoneal dialysis. Data from the Southeast, South, and Midwest regions were presented separately; the ones from the North and Northeast regions were combined to keep the balance among the other regions due to their lower participation.

Study variables comprised demographics (gender, age, skin color, and education level), CKD etiology, dialysis vintage, funding, and biochemical parameters. For the monthly collected variables such as serum phosphate (P), total serum calcium (Ca), hemoglobin (Hb), and dialysis urea reduction rate (URR), numbers represent the mean of at least three results of the year; for serum albumin, which was collected once every three months, a minimum of two results were computed. Participants were calssified as having hyperphosphatemia (P > 5.5 mg/dL), hypercalcemia (Ca > 10.5 mg/dL), anemia (Hb < 10 g/dL), inadequate dialysis (URR < 65%), and hypoalbuminemia (Alb < 3.5 g/dL) based on previously recommended cut-off values^6–8^.

We analyzed the frequency with which physicians prescribed erythropoietin, intravenous (IV) iron, sevelamer, and calcium carbonate. Drug prescriptions were expressed as a percent of total months with information. For example, for a participant with 10 months of available information and four months of prescription, drug prescription was 40%. A minimum of 6 months of information in the database was required to calculate drug prescription.

### Statistical Analysis

Variables are reported as means and standard deviations, median and interquartile, or percentages, as appropriate. Means were compared using one-way ANOVA complemented by the Tukey test or Kruskall-Wallis ANOVA complemented by Dunn test. Differences between categorical variables were assessed by the chi-square test. We used the Southeast region as reference.

The SPSS Statistics for Windows version 21.0 was used to analyze the data. P-values <0.05 were considered significant.

## Results

**Figure 1.**
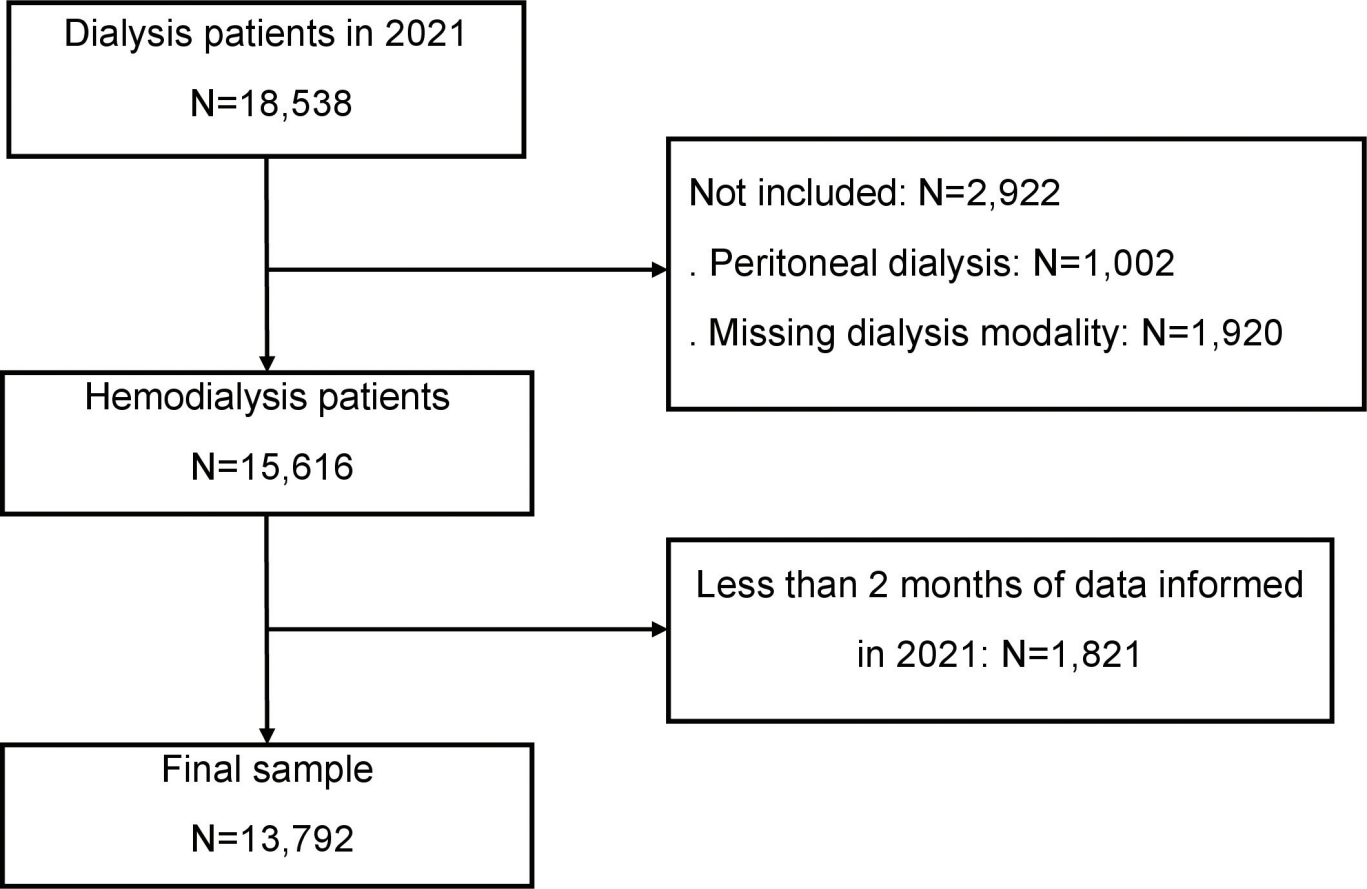
Flowchart of study participants.

A total of 18,538 dialysis patients were identified in 2021, and 13,792 met the inclusion criteria (Figure 1).

The general characteristics of our sample and comparisons across the different geographic regions are shown in Table 1. Patients from the Southeast comprised almost 60% of our sample. Almost half were elderly. Most of the included participants were non-white males with more than eight years of formal education. Hypertension was the most prevalent CKD etiology. The median dialysis vintage was 2.5 years, and the main source of dialysis funding for participants was public (67.5%). Except for gender, all other variables had a different distribution across regions. Patients from all other regions were younger than those from the Southeast. Compared to the Southeast, participants from the South and Midwest had lower educational level. In addition, the South and North/Northeast had a higher prevalence of diabetes and of participants with publicly funded HD treatment than the Southeast.

**Table 1.** General characteristics of the total sample and across geographic regions

	Total N = 13,792	Southeast N = 8,207 (59.5%)	South N = 2,990 (21.7%)	Midwest N = 820 (5.9%)	North/Northeast N = 1,775 (12.9%)
Gender male, N (%)	8,072 (58.5)	4,789 (58.4)	1,725 (57.7)	500 (61.0)	1,061 (59.8)
Age, years Percent > 60 years old	57.9 ± 16.0^a^ 48.9%	58.6 ± 16.3 49.9%	57.5 ± 15.3** 49.1%	56.4 ± 15.3** 44.3%**	56.1 ± 15.9** 45.7%**
Dialysis vintage, months	31 (11–66)^b^	34 (12–68)	27 (8–63)**	34 (11–67)	28 (10–61)**
White skin color, N (%)	6,596 (47.8)	3,596 (43.5)	2,322 (77.7)**	317 (38.7)**	391 (22.0)**
≥8 years at school, N (%)	10,792 (73.6)	6,195 (75.5)	2,007 (67.1)*	588 (71.7)*	1,355 (76.3)
CKD Etiology, N (%) Hypertension Diabetes Glomerulonephritis Polycystic kidney disease Other Unknown	3,057 (27.1) 2,684 (23.7) 1,135 (10.0) 566 (5.0) 3,160 (27.9) 723 (6.4)	2,077 (29.4) 1,438 (20.4) 596 (8.4) 344 (4.9) 2,260 (32.0) 343 (4.9	456 (20.7)** 641 (29.1)** 291 (13.2)** 144 (6.5)** 411 (18.7)** 256 (11.6)**	152 (22.2)** 135 (19.7) 113 (16.5)** 38 (5.5) 231 (33.7) 17 (2.5)**	382 (27.4) 470 (33.8)** 137 (9.7) 40 (2.9) 258 (18.5)** 107 (7.7)**
Public funding, N(%)	9,282 (67.5)	5,050 (61.7)	2,561 (87.7)**	524 (64.5)	1,147 (64.8)*
Incidents, N(%)	1,396 (10.1)	614 (7.5)	443 (14.8)**	111 (13.5)**	228 (12.8)**

^a^Mean ± S.D.; ^b^Median (range); *P < 0.05 vs. Southeast region; **P < 0.01 vs. Southeast region.

Regarding biochemical parameters, significant differences were found between the other regions and the Southeast for every analyzed parameter (Table 2). The prevalence rate of hyperphosphatemia, for instance, was 2.0 and 1.4 times higher in the South and North/Northeast regions, respectively. Hypercalcemia in the South and Midwest regions was 1.8 and 2.1 times higher, respectively. The Southeast had the highest prevalence rate of anemia (32.7%), with the Midwest having close to half of that. The Southeast had the lowest prevalence rate of inadequate URR (<65%) (13.9%), with all other regions having a significantly higher rate. Regarding hypoalbuminemia, the studied population had a prevalence rate of 10.9%, with the Midwest (9.2%) and North/Northeast (7.0%) showing a lower prevalence rate than the Southeast (11.6%).

**Table 2.** Biochemical results of the total sample and across geographic regions

	N	Data availability (Mo.)	Total	Southeast	South	Midwest	North/ Northeast
Serum phosphate, mg/dL >5.5 mg/dL (%)	12,038	8 (5–10)^b^	4.8 ± 1.3^a^ 26.9	4.7 ± 1.2 21.9	5.3 ± 1.4** 41.2**	4.6 ± 1.2 20.2	4.9 ± 1.3** 29.7**
Serum calcium, mg/dL >10.5 mg/dL (%)	11,420	9 (5–10)	8.7 ± 0.7 1.3	8.5 ± 0.7 1.0	8.9 ± 0.7** 1.8**	8.9 ± 0.7** 2.1*	8.8 ± 0.7** 1.4
Hemoglobin, g/dL <10 g/dL (%)	12,119	9 (5–10)	10.5 ± 1.6 31.5	10.5 ± 1.6 32.7	10.5 ± 1.5 30.6	10.9 ± 1.3** 20.8**	10.6 ± 1.6 32.5
Urea reduction rate, % <65% (%)	11,402	9 (5–10)	71 ± 8 18.1	72 ± 8 13.9	69 ± 8** 24.8**	72 ± 10 22.8*	69 ± 8** 22.9**
Serum albumin, g/dL <3.5 g/dL (%)	9,842	3 (2–4)	3.8 ± 0.4 10.9	3.8 ± 0.3 11.6	3.9 ± 0.4** 11.3	3.8 ± 0.3 9.2*	4.0 ± 0.4** 7.0**

Mo.: months; ^a^Mean ± S.D.; ^b^Median (range); *P < 0.05 vs. Southeast region; **P < 0.01 vs. Southeast region.

**Figure 2.**
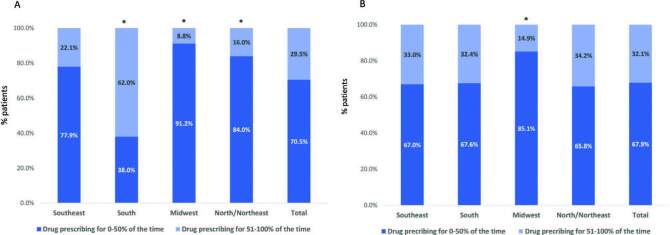
Percent of participants receiving phosphate binders in the different geographical regions: calcium carbonate (Panel A); sevelamer (Panel B). N = 9,375; median number of months with available data: 10 (9–11); *P < 0.01 vs. Southeast region.

The prescription of phosphate binders is showed in Figure 2. In the whole population, sevelamer was prescribed for 0-50% and 51-100% of the time to 67.9% and 32.1% of the participants, respectively. Sevelamer was significantly less used by patients from the Midwest than in those from the Southeast. For calcium carbonate, all regions had significantly different prescription frequency compared to the Southeast, where 22.1% were supplemented for more than 50% of time. In the South, Midwest, and North/Northeast, corresponding numbers were 62.0%, 8.8%, and 16.0%, respectively.

**Figure 3.**
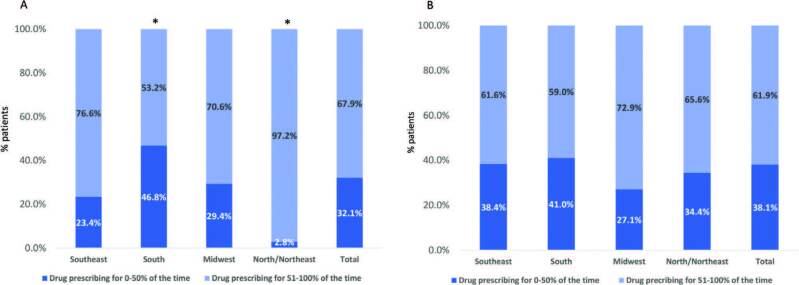
Percent of participants receiving erythropoietin (Panel A) and intravascular iron (Panel B) in the different geographical regions. N = 2,682; median number of months with available data: 10 (8–10); *P < 0.01 vs Southeast region.

Erythropoietin and IV iron prescriptions in people with anemia are shown in Figure 3. The prevalence of patients receiving erythropoietin for more than half of the assessed time was 76.6% in the Southeast region; corresponding figures for the South and North/Northeast were 53.2% and 97.5%, respectively. For IV iron prescription, no significant difference was observed across regions. The percent of participants receiving IV iron for less than 50% of the time in the study population was 38.1% and for more than 50% of the time, 61.9%.

## Discussion

In this retrospective analysis of a large Brazilian chronic HD population, we found differences in demographics, several laboratory parameters and selected medication prescriptions across Brazilian geographic regions.

The geographic distribution of our sample differed from that of the Brazilian general population (Southeast: 59.5 vs. 42%; South: 21.7 vs. 14%; Midwest: 5.9 vs. 7% North/Northeast: 12.9 vs. 36%, respectively). The over-representation of patients from the South and Southeast is due to their higher voluntary participation in the registry.

Patients from the Southeast and South had a higher percentage of elderly (>60 years), corroborating results from a previous large cohort of Brazilian PD patients^4^ and current national demographics^9^. Compared with national population data, included patients had a higher prevalence of whites (47.8 vs. 42.7%)^10^ and a higher percentage of people with complete elementary school (≥ 8 years of schooling; 73.6 vs. 61.4%)^11^. Both results can be attributed to the over-representation of participants from the Southeast and South. These two geographic regions have a higher predominance of European colonization and better social and economic development^2^.

Hypertension and diabetes were the main CKD etiologies of participants, although in lower prevalence rates than those reported in the 2021 Brazilian Dialysis Survey (BDS) (27.1 and 23.7% versus 32 and 30%, respectively)^3^. We can not rule out misclassification of the primary renal diagnosis informed in the BDR as it is generally based on the patient’s history and not confirmed by renal biopsy. This uncertainty is also commonly observed in other registries (USRDS).

The percentage of patients whose dialysis was funded by private health insurance was higher in our sample than in the 2021 BDS, which comprised data from approximately 30% of all people undergoing chronic dialysis in our country (32.5 vs. 18.2%, respectively)^3^.

Surprisingly, comparisons of demographic characteristics across regions show that participants from the South had a lower educational level than those from similar and less developed regions. The most likely explanation is the higher prevalence of patients with public dialysis funding in our sample, reflecting the lower social and economic levels of the patients. Based on comparisons with the 2021 BDS, study participants with private health insurance are over-represented in all regions except the South.

Participants from the South had the highest mean serum phosphate levels and the highest prevalence of hyperphosphatemia, which was 50 to 100% higher than in other regions. Dietary habits in the South are a potential factor in this regard. A small study that compared the frequency of intake of 33 phosphate-containing foods between HD patients from the South and the North found that patients from the South consumed 14 of these foods more frequently, including dairy products and processed meat^12^, in line with the results of the national dietary survey^13^.

One of the strategies used to treat hyperphosphatemia is the use of oral phosphate binders^14^. Although we did not observe a higher frequency of prescription of sevelamer in patients from the South, this was true for calcium carbonate. Sixty-two percent of participants used it over 50% of the time, against 9 to 22% of participants in the other regions.

The prevalence of anemia in the total sample (31.5%) was slightly higher than that reported in previous publications of the BDS (27–30%)^15^ and slightly lower than a previous analysis from the same registry (33.1%)^5^. The prevalence rates found were similar across regions, except for the Midwest, where 20.8% of patients had low hemoglobin levels (<10 g/dL). Regarding medications, almost all participants from the North/Northeast (97%) with anemia had a prescription of erythropoietin for more than 50% of the assessed time, a number markedly higher than the 53% of the South, 77% of the Southeast, and 71% of the Midwest. The public health system provides erythropoietin free of charge to patients, and its dispensing is subject to local regulations. It is known that in some states patients are actually given the prescribed dose, while in other states, patients receive less medication than indicated due to government restrictions and bureaucracy. We wonder if this fact may have impacted drug prescription. No differences were observed in the use of IV iron across regions. There are several factors involved in the prescription of EPO and IV iron and in anemia assessment that are beyond the scope and require further investigation.

Concerning dialysis adequacy, the region with the best results was the Southeast, with only 13.9% of patients with inadequate post-dialysis URR, whereas the figures for the other states were between 23–25%. Of note, these prevalence rates are far better than the 38.5% of inadequate URR found in a report of this registry comprising 24,930 prevalent and incident patients between 2011–2017^5^. In the last reports of the BDS, Kt/V <1.2 was the indicator used for dialysis inadequacy and was found in 18-20% from 2016 to 2019^15^. The URR, although not as precise as the Kt/V, has long been accepted in the international literature as a useful and practical measure for assessing dialysis adequacy, particularly in large databases.

Dialysis adequacy is influenced by several factors, including dialysis-related parameters (session frequency and duration, type of HD membrane) and patient characteristics (residual renal function, body size, fluid overload, etc.)^7^. Further studies addressing this aspect are necessary to explain this finding.

The overall prevalence of hypoalbuminemia (10.6%) was lower than in the last BDS report (14–16%)^15^. Midwest and North/Northeast patients were less likely to have low serum albumin levels compared with Southeastern participants. Serum albumin is influenced by inflammation, comorbidity, liver failure, nutritional status, volume expansion, and urinary or dialysate protein losses^16^. The lack of investigation of these parameters prevents us from finding plausible explanations for these differences.

As study limitations, we highlight the low participation of dialysis centers from the North/Northeast, the lack of more detailed information about comorbidities and some clinical and laboratory parameters such as serum calcium adjustment for serum albumin, and EPO and iron use indications. Also, the study has the hurdles inherent to the retrospective study design. Since the data collection took place in 2021, our findings may have been influenced by the COVID-19 pandemic, although we do not have information about medication shortage in this period. As strengths, we should mention the large number of participants from accross the country and the report of multiple biochemical analyses and medication prescriptions. In addition, this report provides useful information for people and agencies involved in renal replacement therapy, such as industry, government, policy makers, and health professionals, enabling regional comparisons and care planning.

In conclusion, we found differences in demographics, clinical-laboratory parameters, and drug prescriptions of chronic dialysis patients across Brazilian geographic regions. Highlighting these inequalities is pivotal, particularly in a large country like Brazil. Some of these differences reflect the socio-demographic diversity of our country, while others deserve further elucidation.
